# Heat Stress in *Pinus halepensis* Somatic Embryogenesis Induction: Effect in DNA Methylation and Differential Expression of Stress-Related Genes

**DOI:** 10.3390/plants10112333

**Published:** 2021-10-29

**Authors:** Cátia Pereira, Ander Castander-Olarieta, Ester Sales, Itziar A. Montalbán, Jorge Canhoto, Paloma Moncaleán

**Affiliations:** 1 Center for Functional Ecology, Department of Life Sciences, University of Coimbra, 3000-456 Coimbra, Portugal; catia.pereira@student.uc.pt; 2 Department of Forestry Science, NEIKER-BRTA, 01080 Arkaute, Spain; acastander@neiker.eus (A.C.-O.); imontalban@neiker.eus (I.A.M.); 3 Departament of Ciencias Agrarias y del Medio Natural, Instituto Universitario de Ciencias Ambientales, Universidad de Zaragoza, Escuela Politécnica Superior, 22071 Huesca, Spain; esales@unizar.es

**Keywords:** Aleppo pine, conifers, *DEHYDRATION INDUCED PROTEIN 19*, epigenetics, priming, *SUPEROXIDE DISMUTASE* [*Cu–Zn*], 5-hydroxymethylcytosine, 5-methylcytosine

## Abstract

In the current context of climate change, plants need to develop different mechanisms of stress tolerance and adaptation to cope with changing environmental conditions. Temperature is one of the most important abiotic stresses that forest trees have to overcome. Recent research developed in our laboratory demonstrated that high temperatures during different stages of conifer somatic embryogenesis (SE) modify subsequent phases of the process and the behavior of the resulting ex vitro somatic plants. For this reason, Aleppo pine SE was induced under different heat stress treatments (40 °C for 4 h, 50 °C for 30 min, and 60 °C for 5 min) in order to analyze its effect on the global DNA methylation rates and the differential expression of four stress-related genes at different stages of the SE process. Results showed that a slight decrease of DNA methylation at proliferating embryonal masses (EMs) can correlate with the final efficiency of the process. Additionally, different expression patterns for stress-related genes were found in EMs and needles from the in vitro somatic plants obtained; the *DEHYDRATION INDUCED PROTEIN 19* gene was up-regulated in response to heat at proliferating EMs, whereas *HSP20 FAMILY PROTEIN* and *SUPEROXIDE DISMUTASE* [*Cu–Zn*] were down-regulated in needles.

## 1. Introduction

As long-lived sessile organisms with complex life cycles, plants need to develop different mechanisms of protection and adaptation for a broad range of biotic and abiotic stresses in order to maximize growth, reproduction, and survival [1,2]. As well as genetics, epigenetics has become an emerging and promising research field to understand tree phenotypic plasticity and adaptive responses [3,4,5,6,7].

Alterations in epigenetic marks are reversible enzyme-mediated modifications of DNA and/or associated histones that regulate transcriptional activity of genes as well as their sequences [8,9,10]. The most studied epigenetic mark is DNA methylation because of its stability, its incidence in both plants and mammals, and its influence on gene expression and genome structure regulation [11].

Most epigenetics marks are reverted when the environmental constraints that triggered them are no longer present. However, higher plants appear to be able to retain some “stress memory” or “stress imprinting”, since a first stress exposure often leads to an enhanced resistance to a later stress [12,13]. What is also known as “priming” or “hardening”, prior exposure to the eliciting factors leads to a faster and stronger induction of basal resistance mechanisms (or greater tolerance) against them. In some cases, crosstalk among different stimuli can happen, leading to multiple stress memory attainments (cross-priming) [14]. Heat is sometimes accompanied by other stresses, such as drought, and recent studies showed that both stresses have overlapping roles [15,16,17].

Variations in epigenetic marks were revealed to be involved in morphological and physiological changes in trees in a large number of processes, including embryogenesis, organ maturation, phase change, and bud set or burst [18,19,20,21]. Moreover, epigenetic regulation plays a critical role in modulation of multiple aspects of plant development through the adjustment of gene expression in response to environmental factors [8].

Somatic embryogenesis (SE) is a worldwide studied biotechnology tool that allows large-scale propagation for many conifers [22]. The first report of SE in Aleppo pine was carried out in our laboratory [23]. Later, a subsequently developed experiment showed that changes in temperature and water availability at the induction phase of SE affects the success of the process in this species [15]. Considering those results, we focused on the application of higher temperatures (40 °C (4 h), 50 °C (30 min), and 60 °C (5 min)) at the initial stage of SE to see if a “priming” effect could be obtained. It was already found that this stress application during SE induction can modulate the morphology and hormonal profiles of embryonal masses (EMs) as well as the efficiency of the process itself [24].

Notwithstanding that stress affects plants at different levels and several defense mechanisms are activated, it leads to the accumulation of reactive oxygen species (ROS) that can reach toxic levels and cause cell damage and death [25]. To avoid this, plants have evolved antioxidant machinery consisting of enzymatic components, such as superoxide dismutase [26]. Additionally, as previously mentioned, heat is sometimes accompanied with drought, and the dehydration-induced 19 family of proteins appears to be involved in the response to both stresses [27,28]. Finally, the induction of heat shock proteins (HSPs) seems to be essential to facilitate continued homeostasis and survival against heat stress [29,30].

In this sense, in the present study, the effects of different temperature treatments applied during the initial stage of Aleppo pine SE on the epigenetic patterns were evaluated. For this reason, levels of cytosine residues 5-methylcytosine (5mC) and 5-hydroxymethylcytosine (5hmC), both at proliferating EMs and at needles from the in vitro somatic plants produced, were measured. At the same time, the expression of stress-related genes involved in the defense mechanisms mentioned above was analyzed to assess if the initial heat stress triggered long-lasting modifications at the transcriptome along the different stages of the process.

## 2. Results

### 2.1. Global DNA Methylation/Hydroxymethylation Analysis

No statistically significant differences were found regarding global DNA methylation rates (%) between samples from the different induction temperature treatments applied at the initiation stage of *P. halepensis* SE (Table S1).

Regarding the results obtained for EMs, the treatment that presented the lowest levels of 5mC was 60 °C (5 min) (37.52%), followed by the control (23 °C) (38.01%), and the highest methylation rate was obtained at 50 °C (30 min) with a difference of 3.3% with respect to the lowest (40.82%) (Table S2). The values obtained in needles were very similar between treatments (Table S2). All samples presented high rates of global DNA methylation (between 37.52 and 41.56%) (Table S2).

The detection of 5hmC was not possible in proliferating EMs, and it was only achieved, at very low levels, in five out of twelve analyzed samples of needles from in vitro somatic plants. Thus, no further analysis was carried out concerning hydroxymethylation data. Even so, it should be noticed that three of the five detected values at needles corresponded to the samples from the control (23 °C).

### 2.2. Relative Expression of Stress-Related Genes

The different induction temperature treatments applied during the initial stage of *P. halepensis* SE led to changes in the relative expression patterns of the stress-related genes with respect to the control (23 °C).

**Figure 1:**
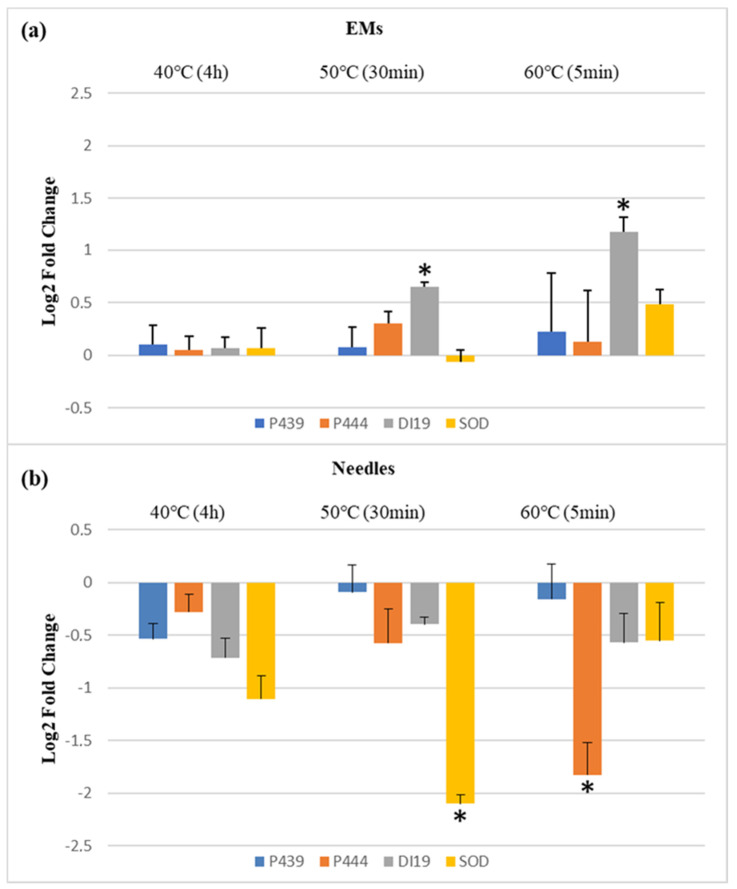
Fold-relative gene expression of four stress-related genes (*P439*, *P444*, *DI19*, and *SOD*) between *P. halepensis* samples induced under different temperature treatments (40 °C, 4 h; 50 °C, 30 min; 60 °C, 5 min) from (**a**) proliferating embryonal masses (EMs); (**b**) needles from in vitro somatic plants. Data are presented as mean values ± SE, and * represents statistically significant differences at *p* < 0.05 of different temperature treatments with respect to the control (23 °C).

The results found in proliferating EMs showed that statistically significant differences were found for the relative expression of *DEHYDRATION INDUCED PROTEIN 19 (DI19*) (Table S3); in both 50 °C (30 min) and 60 °C (5 min) treatments, despite the lower fold change obtained (Figure 1a), the relative expression of this gene was considerably higher than in the control treatment (23 °C). *CHLOROPLAST SMALL HEAT PROTEIN* (*P439*), *HSP20 FAMILY PROTEIN* (*P444*), and *SUPEROXIDE DISMUTASE* [*Cu–Zn*] (*SOD*) were slightly higher expressed in samples induced at higher temperatures when compared to the control, except for *SOD* at the 50 °C treatment (Figure 1a), but these differences were not significant.

In contrast, in needles from in vitro somatic plants, statistically significant differences were found for the relative expression of *P444* and *SOD* genes. A gradual decrease of *P444* relative expression was observed when priming temperature increased, being significantly repressed compared to control (23 °C) for the 60 °C treatment. Expression of the *SOD* gene was lower in relation to the control (23 °C), but significant differences were only detected in samples coming from the 50 °C treatment, which attained the highest fold change (Figure 1b). *P439* and *DI19* were slightly down-regulated at samples induced at higher temperatures when compared to the control (23 °C), but these differences were not significant. In conclusion, induction of SE at higher temperatures resulted in different relative expression patterns of these stress-related genes in proliferating EMs and in needles from in vitro somatic plants. At proliferating EMs, the stress-related genes studied were generally overexpressed in primed plant material, whilst in the in vitro somatic plants obtained, their expression was lower in primed than in control.

## 3. Discussion

Accumulating data have shown that epigenetics changes are involved in many physiological processes, and the understanding of these mechanisms is crucial for forest tree management and breeding in the context of climate change [31,32,33]. One of the most important epigenetic marks is DNA methylation. It occurs through the addition of a methyl group at position 5 of the pyrimidine ring of cytosine in the CG, CHG, and CHH (where H = A, T, or C) contexts [34,35], and understanding how its levels change under stressful conditions can lead to a better knowledge of the plant response to environmental changes [11,36]. Considering the use of epigenetic variations for breeding applications relies on their transmission features, and since 5mC patterns can be transmitted through mitosis as well as meiosis, this DNA methylation mark could be valuable in all crops regardless of their propagation method [37]. In this sense, the concentrations of 5mC and 5hmC were assessed in order to perform a global DNA methylation study in samples from proliferating EMs and needles from in vitro somatic plants of Aleppo pine induced under high temperatures.

No statistically significant differences in methylation status were found between the induction treatments applied. Nonetheless, at proliferating EMs, a difference of 3.3% was found between the 60 °C (5 min) treatment that presented the lowest concentrations of 5mC with respect to the highest methylation rate obtained at 50 °C (30 min). In *Pinus nigra,* specific DNA methylation levels were analyzed in tissues with different embryogenic potentials, and the lowest levels were found in those with higher embryogenic capacity [38]. In *Coffea canephora* indirect SE, 5mC rates were stable during cell differentiation, and a significant increase occurred during somatic embryos regeneration [39]. Several studies suggest that DNA methylation is critical for SE success, and it is common to find lower values in embryogenic tissues with respect to the non-embryogenic tissues [5,40,41]. Taking this into account and correlating the results presented here with the ones obtained in a previous study, where the effect of *Pinus halepensis* SE induction under high temperatures on the efficiency of the process was assessed, we can reaffirm this plausible hypothesis. The highest number of somatic embryos produced was obtained in EMs from the 60 °C (5 min) treatment (a mean of 317.4 somatic embryos per gram) followed by those from the control; the lowest number was gained when a priming treatment of 50 °C (30 min) was applied (a mean of 135.2 somatic embryos per gram) [24].

Considering the effect of heat stress on the variation of DNA methylation levels, different species and cell types display different responses. In a similar study, performed with *P. radiata,* the same decrease in samples induced at the highest temperature of 60 °C (5 min) compared to the control temperature and the 40 °C (4 h) treatment was found [42]. On the contrary, in Norway spruce, seedlings originating from a warm embryonic environment [43] and the exposure of *Arabidopsis* plants to heat [44] resulted in an increase of global DNA methylation. Interestingly, also in *Arabidopsis*, DNA methylation increased during heat stress, followed by the reduction of DNA methylation levels after transfer to the control conditions [36].

Needles presented slightly higher concentrations of 5mC relative to EMs, and the global DNA methylation values were stabilized between treatments at this stage. This is in accordance with the fact that a gradual increase in DNA methylation throughout ageing was previously reported for several forest trees [45,46]. In *P. radiata* needles from field trees, DNA methylation increased with both ageing and phase change [47]. In contrast, 5mC levels in proliferating EMs and needles from one-year-old somatic plants of *P. radiata* were similar [42].

The 5hmC was detected in needles from in vitro somatic plants of Aleppo pine. As reviewed in [48], numerous studies indicate that 5hmC acts not only as an intermediate during 5mC demethylation but also plays important roles during maintenance of pluripotency in animal embryonic stem cells; however, in plants the roles of 5hmC during development are still unknown. The first discovery of its presence in conifers was in Norway spruce [49], and it was found that its concentration can fluctuate not only at different tissues but also at different temperatures in *P. radiata* [42].

The differential expression of stress-related genes (*P439*, *P444*, *SOD*, and *DI19*) in primed plant material was also assessed in this study. To respond and adapt to different stresses, plants have developed a complex of molecular mechanisms by modulating the expression of a specific set of genes [11,33,50,51], and we found that differential expression patterns changed between the two stages of the propagation process analyzed. 

Regarding the relative expression of the stress-related genes in proliferating EMs, statistically significant differences were only found for *DI19*. A gradual increase at the relative expression of this gene along the higher temperature treatments was found and in EMs initiated at 50 °C (30 min) and 60 °C (5 min) was considerably higher than in the control (23 °C). The DI19 family of proteins is a novel type of Cys2/His2 zinc-finger proteins involved in the response to several abiotic stresses, especially drought [52]. In *Populus simonii*, it was found that there is an overlapping heat–drought response [53], and our results suggest that *DI19* overexpression is involved in the abiotic stress response in *P. halepensis*. In accordance, in rice, the overexpression of the DI19-4 resulted in significantly increased tolerance to drought stress [54], and in *Arabidopsis*, DI19-1 overexpressing lines presented higher tolerance to drought stress than the wild-type lines [55]. On the other hand, in *P. radiata* emerging EMs induced at higher temperatures, *DI19* initially presented similar levels to control; however, after 4 weeks, actively proliferating EMs induced at 40 °C (4 h) were significantly underexpressed [42]. In another study in *Arabidopsis*, transgenic lines with DI19–3 overexpression were more sensitive to salinity and drought than wild-types [52]. It appears that different protein members of this family, despite being clearly involved in stress response mechanisms, may have different functions unknown yet.

No differences were obtained in proliferating EMs for *P439*, *P444*, and *SOD*. However, needles from in vitro grown somatic plants showed statistically significant differences in transcript abundance for both *P444* and *SOD* genes. The relative expression of these genes was lower in plants coming from EMs initiated at high temperatures when compared to the control plants. Heat stress severely affects the stability of various cellular components, causing a state of metabolic imbalance and a cascade of cellular reactions. This disruption of the steady-state flux of cellular metabolites usually leads to the accumulation of toxic products, such as ROS [50,53]. Furthermore, HSPs are closely involved in cellular protection, its structures and responses to heat stress being highly conserved amongst several organisms [29,56]. According to this, in maritime pine, when heat priming was performed at immature megagametophytes through SE, similar levels of expression were observed in primed and control EMs for *HSP70* and *SOD*, but the expression of *HSP70* at the derived in vitro somatic plants was higher in control plants [57]. In *P. radiata,* the gene coding for a heat shock protein (*HSP20*) was down-regulated in proliferating EMs and somatic plants coming from EMs initiated at high temperatures [42]. In contrast, *P. radiata* one-year-old seedlings subjected to heat treatments showed significantly higher short-term expression of *P439* and *P444* [58]. Different sampling times, as well as different tissues, seem to lead to different relative expressions of stress-related genes. Taking this into account, we suggest that in *P. halepensis* stress-related gene overexpression may happen during and/or shortly after the heat stress occurs. With time and at different tissues, the priming effect leads to their stabilization and their lower expression relative to controls.

Finally, it is important to note that different patterns of stress-related genes relative expression have been observed. In this sense, *DI19* presented a gradual increase along the higher temperatures at EMs and *P444* a gradual decrease in the needles from in vitro somatic plants. In its turn, *SOD* was significantly repressed in needles sampled in plants derived from priming at 50 °C (30 min), while those from plants primed at 60 °C (5 min) showed expression rates similar to non-primed plants. When the effect of these temperature treatments in EMs endogenous cytokinin (CK) profiles was assessed in a previous work on this species, diverse patterns were also found for different CKs [24]. Those results were related to the different induction times between treatments and the concept that for some CKs, temperature treatments acted as short or mild stress, while for others they were sensed as a prolonged or more severe stress. In *Arabidopsis*, heat stress has been connected with fluctuations in the endogenous levels of CKs and ABA that seem to be involved in HSPs regulation [59]. It is possible that an interaction between CKs and the regulation of other stress-related genes, in response to heat stress, also occurs in *P. halepensis*.

## 4. Materials and Methods

### 4.1. SE Temperature Experiment and Plant Material Collection

Induction of *Pinus halepensis* EMs under different temperatures was performed as described in [24]. Briefly, one-year-old green female cones, enclosing immature seeds of *P. halepensis* from five open pollinated trees were used; storage and preparation of plant material was the same as described in [60]. Whole megagametophytes were placed horizontally on DCR initiation medium [61]. For temperature treatments, closed Petri dishes containing initiation medium were preheated for 30 min, and immature megagametophytes were cultured at 40, 50, and 60 °C for 4 h, 30 min, and 5 min, respectively. As control, 23 °C was used, and, after the application of the different treatments, all explants were kept at standard conditions in darkness. 

After nine weeks on the initiation medium, proliferating EMs were detached from the megagametophyte and transferred to the proliferation medium. This medium had the same composition to that used in the initiation stage, but a higher gellan gum concentration (4.5 g L^−1^). EMs were subcultured every two weeks and kept in the dark.

Following 4 subcultures, fresh tissue from twenty proliferating embryogenic cell lines (ECLs) were immersed in liquid nitrogen and immediately stored at −80 °C until further analysis. Eight/nine ECLs per treatment were selected to maturation at DCR medium supplemented with 60 g L^−1^ sucrose, 75.0 µM abscisic acid, the EDM amino acid mixture [62], and 9 g L^−1^ Gelrite^®^.

For germination, somatic embryos were transferred to Petri dishes containing half-strength macronutrient LP medium [63,64] supplemented with 2 g L^−1^ of activated charcoal and 9.5 g L^−1^ Difco^®^ granulated agar (Becton Dickinson, Franklin Lakes, NJ, USA). Cultures were cultured under dim light for 7 days and afterwards were kept under a 16:8 h photoperiod at 100 µmol m^−2^ s^−1^ provided by cool white fluorescent tubes (TFL 58 W/33, Philips, France). The obtained plantlets were transferred onto fresh medium of the same composition every 4 weeks. During the first 8 weeks, plantlets were cultured in Petri dishes and then transferred to glass culture vessels. After 6 months, needles from in vitro somatic plants from eleven ECLs were immersed in liquid nitrogen and immediately stored at −80 °C until further analysis. 

### 4.2. Global DNA Methylation/Hydroxymethylation Analysis

Genomic DNA extraction and subsequent methylation analysis were performed both on samples from twenty proliferating EMs and needles from in vitro somatic plants from eleven ECLs (comprising five samples of EMs and three samples of needles, per treatment). Previously collected samples were lyophilized, and 15 mg of homogenized lyophilized tissue were used.

DNA extraction and its hydrolyzation were performed as described in [42].

Methylation and hydroxymethylation levels of cytosine were analyzed on a 1200 Series HPLC system coupled to a 6410 Triple Quad mass spectrometer (Agilent Technologies, Santa Clara, CA, USA). The chromatographic separation was performed on a Zorbax SB-C18 column (2.1 × 100 mm, 3.5 µm, Agilent Technologies). The mobile phase was 11% methanol and 0.1% formic acid in water, and 5 µL of samples were injected in the column at a flow rate of 0.1 mL min^−1^. The electrospray ionization source (ESI) was operated in the positive ion multiple reaction monitoring mode (MRM) set to an ion spray voltage of 3500 V, 40 psi for nebulizer, and source temperature at 350 °C. The intensities of specific MH^+^→ fragment ion transitions were recorded (5mC *m*/*z* 242→126, 5hmC *m*/*z* 258→142, and C *m*/*z* 228→112). Identification of cytosine, 5mC, and 5hmC was assessed by injection of commercial standards (5-Methylcytosine and 5-Hydroxymethylcytosine DNA Standard Set, Zymo Research, Irvine, CA, USA) under the same LC-ESI-MS/MS-MRM conditions. The percentage of 5mC and 5hmC at each sample was calculated from the MRM peak area divided by the combined peak areas for 5mC, 5hmC, and cytosine.

### 4.3. Relative Expression of Stress-Related Genes

RNA extraction and further analysis of expression patterns from stress-related genes were performed on samples from sixteen proliferating EMs and needles from in vitro somatic plants from eleven embryogenic lines (comprising four samples of EMs and three samples of needles, per treatment). As initial material, 10 mg of lyophilized tissue, previously grinded for homogenization in a TissueLyser II (Qiajen, Hilden, Germany), were used. The analysis was performed based on the protocol described in [42].

Total RNA extraction was carried out using a plant/fungi total RNA purification kit (Norgen Biotek Corp., Thorold, ON, Canada), and genomic DNA was degraded by using recombinant DNase I (RNase-free, Takara Bio Inc., Shiga, Japan), following manufacturer’s instructions. A Nanodrop^TM^ 2000 was used for RNA quantification, and its integrity was assessed by agar gel electrophoresis.

cDNA was synthesized from 1000 ng of RNA using the PrimeScript RT Reagent Kit (Takara) and random hexamers as primers following the manufacturer’s instructions. Real time PCR amplifications were performed in StepOne Plus (Applied Biosystems, Carlsbad, CA, USA), using a final volume of 20 µL containing 0.8 µM of each primer and 10 µL of SYBR Green I Master mix (Takara Bio Inc., Shiga, Japan) in triplicate for each sample. The PCR conditions were an initial denaturation at 95 °C for 20 s, followed by 40 cycles of 95 °C for 3 s and 60 °C for 30 s. 

Primers previously described [28,58] were used and their efficiencies estimated using the qPCR Efficiency Calculator available at Thermofisher.com, based on the standard curve previously developed with four dilution points for each primer. Analyzed genes as well as primers details are summarized in Table 1.

The relative transcript levels were normalized using ACTIN (ACT), and the relative expression of each gene (R) was calculated on the basis of ΔCt values using the following formula: R = 2^−ΔCt^ [65]. Finally, the fold changes between expression values obtained at control treatment (23 °C) and different temperature treatments were calculated in logarithmic scale.

### 4.4. Statistical Analysis

A one-way analysis of variance, through the application of the non-parametric Kruskal–Wallis test, was carried out to assess the effect of different temperature treatments both for total DNA methylation rates (%) and for genes’ relative expression values. When significant differences were found (*p* < 0.05), Dunn’s multiple comparison test was carried out to find out which treatments were statistically different.

## 5. Conclusions

As far as it is known, this is the first report concerning the effect in DNA methylation and expression of stress-related genes in response to heat stress application during SE induction in Aleppo pine.

Regarding the DNA methylation results, the temperatures treatments applied were not enough to provoke significantly different levels of DNA methylations at the analyzed samples. Nonetheless, it is important to note that the treatment that presented the lowest methylation level was also the one that produced the highest number of somatic embryos (60 °C, 5 min) [24]. It appears that lower levels of DNA methylation/DNA hypomethylation are associated with higher embryogenic capacity and, therefore, with a higher number of somatic embryos produced. 

Concerning the application of high temperatures at the early stage of *P. halepensis* SE at the expression of stress-related genes, long-term changes in the differential expression of stress-related genes, specifically *DI19*, *P444*, and *SOD*, at different stages of SE were found. Despite the fact that the pattern of overexpression of primed EMs and lower expression of primed needles were consistent, it appears that different induction times between treatments had an effect on the relative expression of the stress-related genes studied.

Further analysis concerning the effect of heat stress application during the induction phase of *P. halepensis* SE regarding its effect on metabolomics and protein profiles will be performed. This data can lead to a better understanding of all the mechanisms involved on the heat stress response in this species.

## Figures and Tables

**Table 1:** List of primers used in quantitative real time PCR (qRT-PCR) for relative expression analysis. Names of the genes, forward and reverse primer sequences, and melting temperatures of primers are described.

ID	Name	Forward (5′ → 3′)	Reverse (5′ → 3′)	Tm (°C)
ACT	ACTIN	CACTGCACTTGCTCCCAGTA	AACCTCCGATCCAAACACTG	60
P439	CHLOROPLAST SMALL HEAT PROTEIN	AAGTTGTCGGTTCGAACCCC	CAGAACACCGTCCTCCACAG	62
P444	HSP20 FAMILY PROTEIN	TTTCCGACTTCTTCACGGGG	TTTGACAGTCCCGGCATGTC	62
DI19	DEHIDRATION INDUCED PROTEIN 19	ATAGATGCCCATGCTGTGTAG	CTTCCCTCTGTTCCCACTTG	54
SOD	*SUPEROXIDE DISMUTASE* [*Cu–Zn*]	ACAAAACGGGTGCATGTCAAC	CCCATCCGCTCCTACAGTTAC	66

## Data Availability

It is all original.

## Supplementary Materials

The following are available online at https://www.mdpi.com/article/10.3390/plants10112333/s1, Table S1: One-way analysis of variance for methylation rates (%) detected in *P. halepensis* embryonal masses (EMs) and needles from in vitro somatic plants induced under different temperature treatments (23 °C, 9 weeks; 40 °C, 4 h; 50 °C, 30 min; 60 °C, 5 min). Table S2: Total methylation rates (%) detected in *P. halepensis* proliferating embryonal masses (EMs) and needles from in vitro somatic plants induced under different temperature treatments (23 °C (control); 40 °C, 4 h; 50 °C, 30 min; 60 °C, 5 min). Table S3: One-way analysis of variance for expression of different genes detected in *P. halepensis* embryonal masses (EMs) and needles from in vitro somatic plants induced under different temperature treatments (23 °C, 9 weeks; 40 °C, 4 h; 50 °C, 30 min; 60 °C, 5 min).
