# Supplementary material for: Heat Stress in Pinus halepensis Somatic Embryogenesis Induction: Effect in DNA Methylation and Differential Expression of Stress-Related Genes
Source: Plants (Basel). 2021 Oct 29;10(11):2333. doi: 10.3390/plants10112333 (PMC8622292; doi:10.3390/plants10112333)
Supplement: Supplementary file 1 [file plants-10-02333-s001.zip › plants-1387164-supplement.pdf]

plants

MDPI

Article

Heat Stress in *Pinus halepensis* Somatic Embryogenesis Induction: Effect in DNA Methylation and Differential Expression of Stress-Related Genes

Cátia Pereira [1,2], Ander Castander-Olarieta [2], Ester Sales [3], Itziar A. Montalbán [2], Jorge Canhoto [1,*] and Paloma Moncaleán [2,*]

Supplementary Materials

Table S1. One-way analysis of variance for methylation rates (%) detected in *P. halepensis* embryonal masses (EMs) and needles from *in vitro* somatic plants induced under different temperature treatments (23 °C, 9 weeks; 40 °C, 4 h; 50 °C, 30 min; 60 °C, 5 min).

Kruskal-Wallis	d*f*	X^2 test	*p* value
EMs	3	5.960	n.s.
Needles	3	5.359	n.s.

[1] not statistically significant.

Table S2. Total methylation rates (%) detected in *P. halepensis* proliferating embryonal masses (EMs) and needles from *in vitro* somatic plants induced under different temperature treatments (23 °C (control); 40 °C, 4 h; 50 °C, 30 min; 60 °C, 5 min). Data are presented as mean values ± SE and significant differences at $p < 0.05$ are indicated by different letters.

Methylation (%)	23 °C (Control)	40 °C (4 h)	50 °C (30 min)	60 °C (5 min)
EMs	38.01 ± 0.72 [a]	39.48 ± 1.94 [a]	40.82 ± 0.84 [a]	37.52 ± 0.45 [a]
Needles	40.00 ± 0.21[a]	40.25 ± 1.21 [a]	41.47 ± 0.43 [a]	41.56 ± 0.41[a]

Table S3. One-way analysis of variance for expression of different genes detected in *P. halepensis* embryonal masses (EMs) and needles from *in vitro* somatic plants induced under different temperature treatments (23 °C, 9 weeks; 40 °C, 4 h; 50 °C, 30 min; 60 °C, 5 min).

Kruskal-Wallis	d*f*	X^2 Test	*p* Value
**EMs**			
P439	3	5.286	n.s.[1]
P444	3	6.795	n.s.
DI19	3	35.23	< 0.0001
SOD	3	7.057	n.s.
**Needles**			
P439	3	3.983	n.s.
P444	3	15.75	0.0013
DI19	3	3.297	n.s.
SOD	3	15.42	0.0015

[1] not statistically significant.
